# Awareness of “Predatory” Open-Access Journals among Prospective Veterinary and Medical Authors Attending Scientific Writing Workshops

**DOI:** 10.3389/fvets.2015.00022

**Published:** 2015-08-13

**Authors:** Mary M. Christopher, Karen M. Young

**Affiliations:** ^1^Department of Pathology, Microbiology, and Immunology, School of Veterinary Medicine, University of California-Davis, Davis, CA, USA; ^2^Department of Pathobiological Sciences, School of Veterinary Medicine, University of Wisconsin-Madison, Madison, WI, USA

**Keywords:** education, journal selection, mentoring, open access, publishing, survey

## Abstract

Authors face many choices when selecting a journal for publication. Prospective authors, especially trainees, may be unaware of “predatory” online journals or how to differentiate them from legitimate journals. In this study, we assessed awareness of open-access and predatory journals among prospective authors attending scientific writing workshops; our long-term goal was to inform educational goals for the workshops. We surveyed participants of writing workshops at veterinary and medical schools and an international conference over a 1-year period. The survey included 14 statements for respondents to indicate agreement level on a Likert-like scale and four questions on awareness of resources about predatory journals; respondents also defined “predatory journal.” A total of 145 participants completed the survey: 106 (73.1%) from veterinary schools and 86 (59.3%) graduate students or residents. Fewer faculty (vs trainees) agreed that open access was an important factor in deciding where to publish; faculty and postdoctoral researchers were more likely to expect to pay more to publish in an open-access journal. Most respondents (120/145, 82.7%) agreed/strongly agreed that the decision to accept a manuscript should not be influenced by publication charges, but 50% (56/112) indicated that they “didn’t know” how publishing costs were supported. Of the 142 respondents who answered, 33 (23.0%) indicated awareness of the term “predatory journal”; 34 (23.9%) were aware of the Directory of Open Access Journals; 24 (16.9%) were aware of the Science “sting” article about predatory journals; and 7 (4.8%) were aware of Beall’s list. Most (93/144, 64.5%) definitions of predatory journals described poor but not predatory journal practices, and some respondents misunderstood the term completely. Mentors should help novice authors to be aware of predatory journals and to distinguish between legitimate and illegitimate open-access journals, thus selecting the best journal for their work.

## Introduction

Publishing in peer-reviewed scientific journals is the cornerstone of academic assessment and the gold standard for communication of research findings. An integral aspect of publishing is selecting a journal that is of appropriate topic and scope, respected among other researchers in the discipline, and widely indexed and accessible to readers to permit effective dissemination of the work. Open-access journals have expanded enormously in number and scope during the past 20 years to attract authors keen to give their work prompt and unfettered access (1, 2). Proliferation of online open-access journals has included major journal “brands” published by well-known publishers, such as BMC and PLoS, as well as journals and publishers that lack a legitimate foundation and use online publishing solely for financial (rather than scientific) gain (3–7). Journals have been termed “predatory” when they present a seemingly legitimate face for an illegitimate publication process that lacks basic industry standards, sound peer-review practices, or a solid basis in publication ethics (7). Such journals exploit the pressure to publish and the desire for access and can create confusion on the part of prospective authors and readers.

The term “predatory” journals is not without controversy, in part because online journals range widely in quality and vary in the scientific credentials of the editorial staff, rigor of peer review, types of articles published, policies of the publisher, and quality of the work and the writing. Some journals may reflect a blend of legitimate and illegitimate practices that are difficult to discern or impossible to classify. Authors, especially those with little experience, may find evaluating the quality of journals difficult. In cultures and countries without a robust research infrastructure, the attraction of successful publication in an open-access journal may obscure the need to investigate the legitimacy of a journal. A recent study found that authors who publish in predatory journals have limited publishing experience and often are located in developing countries (8). However, even authors operating in an environment of rigorous research and publication may be unaware of predatory journals, and the recent focus on open access and plethora of open-access journals could obscure the problem.

Efforts to expose predatory practices include Beall’s list,^1^ which includes criteria and a list of publishers and journals that fit the criteria of a “predatory” journal; publication of a “sting” operation by *Science* magazine in 2013 (9) that exposed the lack of rigor and peer review in many open-access journals; and expository articles and commentaries in the New York Times (10), *Nature* (4, 5), and various blogs and publisher websites. Despite the media attention on predatory journals, we have observed – as journal editors and educators who teach scientific writing and publishing across a range of biomedical audiences – that many prospective authors, and even some experienced researchers and editors, are unaware of the challenges involved in selecting a journal. In order to inform our educational goals in conducting scientific writing workshops and in mentoring academic writing, we integrated a survey into our workshops and courses to ascertain the level of awareness of open-access and predatory journals among participants. The results of this survey suggest that additional work is needed not only to increase awareness but also to inform authors of journal processes important to maximizing the quality and distribution of published scientific work.

## Materials and Methods

The survey consisted of 14 statements (2 of which, #4 and #12, were added after the first workshop), for which respondents indicated their level of agreement or disagreement on a Likert-like scale. We also asked four Yes/No questions on whether the respondent was aware of the Directory of Open Access Journals (DOAJ),^2^ Beall’s list, the term “predatory journal”, and the recent (October 2013) article published in *Science* (9) about the “sting” operation involving open-access journals. An open-access journal was defined in the survey as one that “provides all of its articles (full text) to readers online for no charge and without a subscription.” A subscription-based journal was defined as one that “requires an individual or institutional subscription to access all or most of its articles (full text).” Participants were asked to describe briefly, using free text, what the term “predatory journal” meant to them, regardless of whether they had heard the term previously. Respondents also were asked to specify whether they were a graduate student, resident, postdoctoral researcher (postdoc), faculty member, or other. The University of California-Davis (UCD) Institutional Review Board (IRB) Administration determined that administration of the survey did not require prior submission to the IRB because the federal definition of human subjects research was not met (the survey was conducted as part of classroom/course activity to assess the current knowledge of participants and inform course curriculum).

The survey was distributed on paper as part of scientific writing workshops or courses given by one or both of the authors during the 1-year period from November 2013 to October 2014. Formats and venues included a 1-day workshop at the annual meeting of the American Society for Veterinary Clinical Pathology (ASVCP) in Montreal, Canada (November 2013); a 1-day workshop at the UCD School of Veterinary Medicine (December 2013); a 1-unit graduate course at the UCD School of Veterinary Medicine (January 2014 and April 2014); a seminar series at the University of Wisconsin-Madison (UW) School of Veterinary Medicine; a 2-h seminar at the UW School of Medicine and Public Health (March 2014); a 1-day workshop at the UCD School of Medicine, Clinical and Translational Science Center (August 2014); and a 1-day workshop at a Faculty (School) of Veterinary Medicine in southern Europe (Eur-SVM) (September 2014). Respondents attending the ASVCP workshop came from a variety of countries (Australia, Austria, Canada, Germany, Japan, South Africa, Sweden, Switzerland, and the U.S.). At all venues, the survey was distributed and completed prior to presentation or discussion of journal types and selection. Participants were informed that the survey was anonymous, its completion was optional, and results would be shared and used to guide and improve the content of future workshops and courses. When administered in a graduate course, students were told that completion of the survey had no bearing on their grade in the course. Respondents were given ~15 min to complete the survey, which was then collected.

Responses were numbered and results entered into an Excel spreadsheet (Microsoft, Redmond, WA, U.S.). Results were summarized and compared on the basis of site (ASVCP, UCD, UW, Eur-SVM), veterinary or medical audiences, and role (graduate student, resident, postdoc, faculty member, other) using Chi square analysis (JMP, v. 11.2, SAS Institute Inc., Raleigh, NC, U.S.). Differences were considered statistically significant when *P* < 0.05.

Free-text definitions of “predatory journal” were grouped thematically using the general criteria published on Beall’s list for predatory journals.^1^ These criteria have been subject to considerable input and discussion and draw on best practices in scholarly publishing provided by the Committee on Publication Ethics (COPE).^3^

## Results

A total of 145 participants completed the survey (Table 1). Three-fourths of respondents were from veterinary institutions; 11 students in one graduate course were a mixed group from both medical and veterinary schools, so were not included in the analyses comparing veterinary and medical audiences. More than half of the respondents were graduate students and residents. Respondents who indicated their role as “other” included veterinary diagnostic pathologists (*n* = 5), interns (*n* = 3), staff (*n* = 3), and one each of a scientist, career development award recipient, DVM/PhD in private practice, veterinary student, medical student, fellow, and a prospective graduate student doing mentored research. Four participants did not state their role. Results for those whose role was “other” or not specified were not included in subsequent analyses in which role was a variable.

**Table 1:** **Demographic information on writing workshop participants based on role (A) and medical vs veterinary audiences (B) at the various sites**.

**(A)**						

Site	Faculty	Postdocs	Grad students	Residents	Other	Total
ASVCP	8	2	3	12	7	32 (22.0%)
UCD	9	7	35	8	8	67 (46.2%)
UW	3	8	1	4	4	20 (13.7%)
Eur-SVM	1	0	20	3	2	26 (17.9%)
Total	21 (14.4%)	17 (11.7%)	59 (40.6%)	27 (18.6%)	21 (14.4%)	145

**(B)**						

**Site**	**Medical audience**	**Veterinary audience**	**Mixed medical/veterinary**			

ASVCP	0	32	0			
UCD	14	42	11			
UW	14	6	0			
Eur-SVM	0	26	0			
Total	28 (19.3%)	106 (73.1%)	11 (7.5%)			

**Figure 1:**
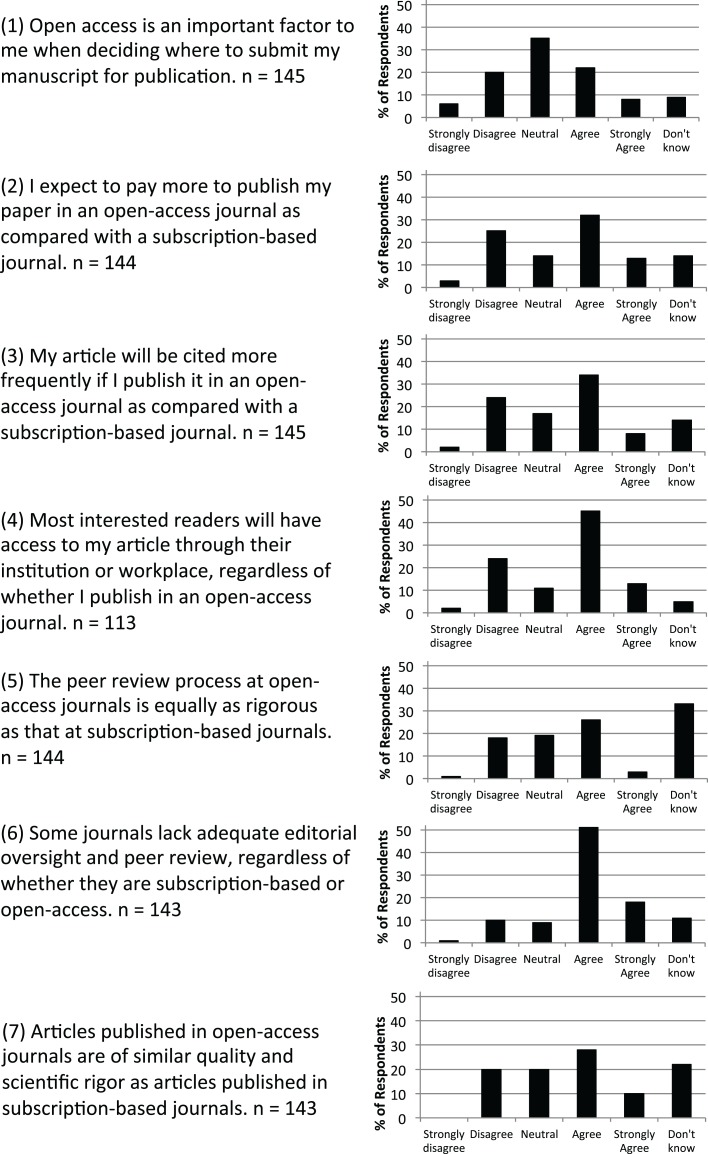

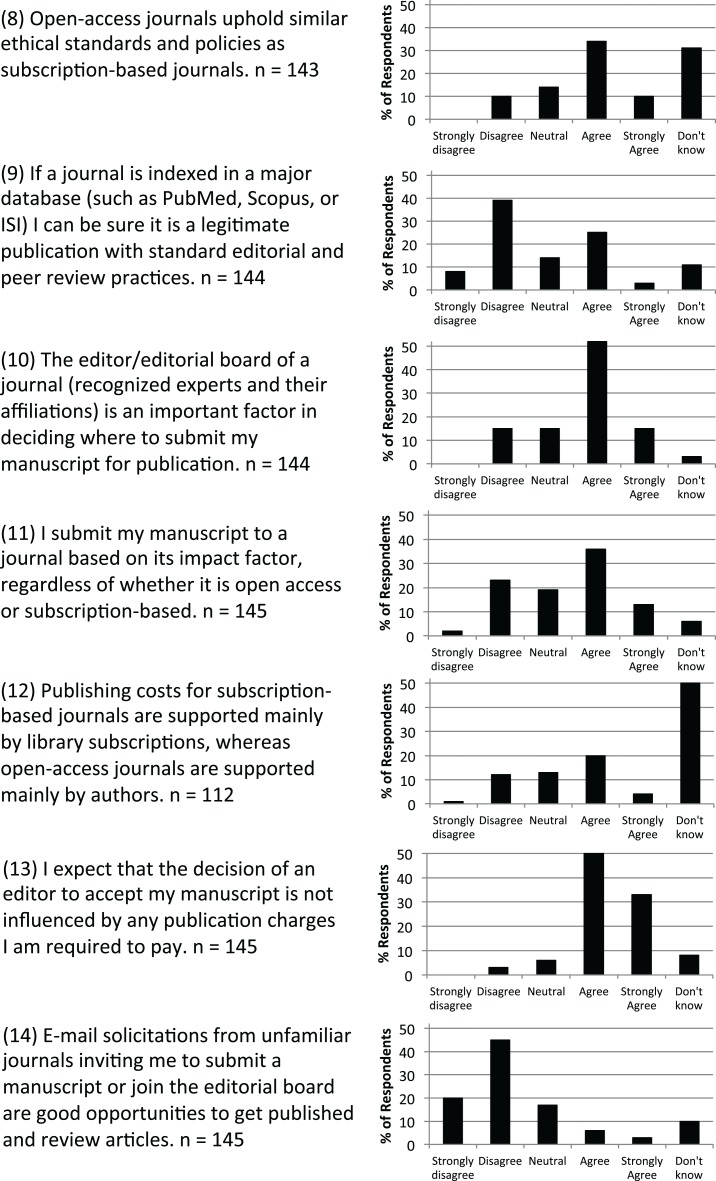
**Level of agreement indicated by respondents to 14 statements about open-access and subscription-based journals**.

Responses were summarized for each of the 14 statements about open-access and subscription journals (Figure 1). Because statements #4 and #12 were added after the first workshop, the number of responses was lower than for other statements. The highest level of agreement was found for statement #13, in which 120/145 (82.7%) respondents agreed or strongly agreed that they expect the editor’s decision to accept a manuscript not to be influenced by publication charges. The highest level of disagreement was for statement #14, in which 94/145 (64.8%) respondents disagreed or strongly disagreed that email solicitations from unfamiliar journals presented good opportunities. The highest proportion of “don’t know” responses (56/112, 50.0%) was for statement #12 (how publishing costs are supported), followed by statements #5 (47/144, 32.6%) and #8 (45/143, 31.4%), which addressed the rigors of the peer-review process and ethical policies. The highest proportion of “neutral” responses (51/145, 35.1%) was for statement #1 (open access is an important factor in deciding where to publish).

**Figure 2:**
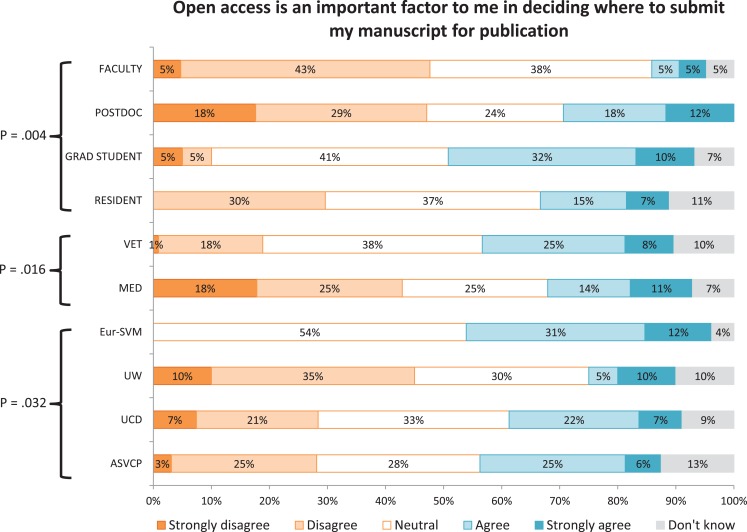
**Differences in levels of agreement based on role, veterinary vs medical audience, and site for survey statement #1**.

**Figure 3:**
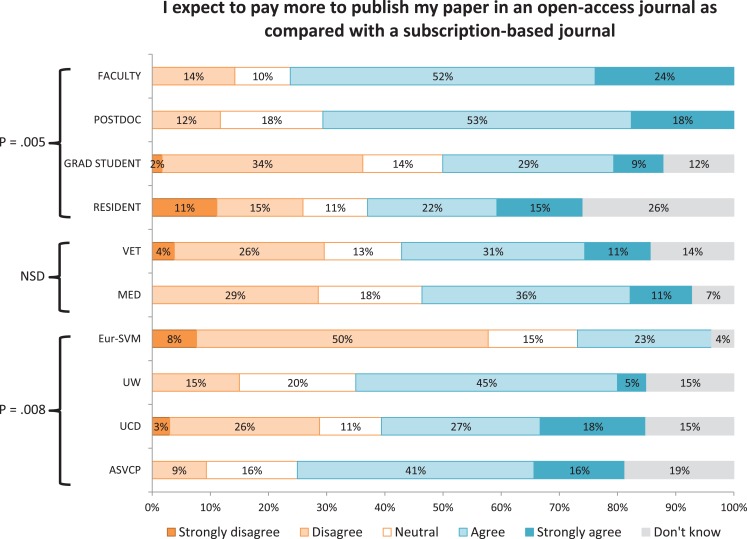
**Differences in levels of agreement based on role, veterinary vs medical audience, and site for survey statement #2**.

**Figure 4:**
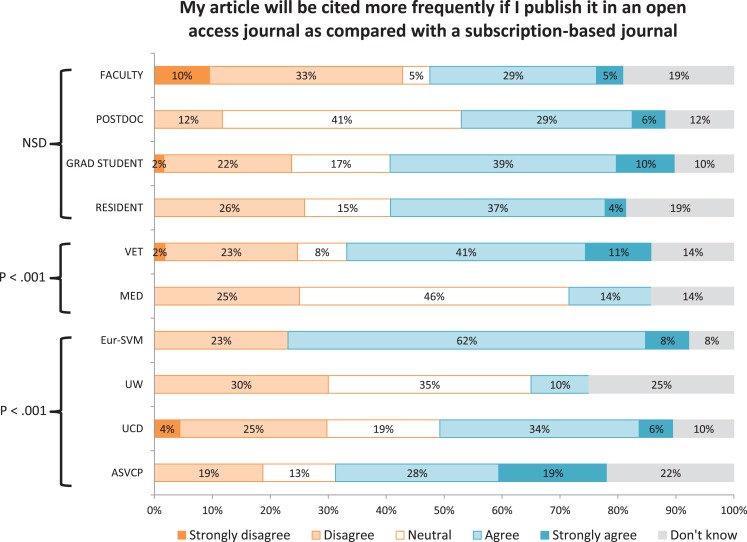
**Differences in levels of agreement based on role, veterinary vs medical audience, and site for survey statement #3**.

**Figure 5:**
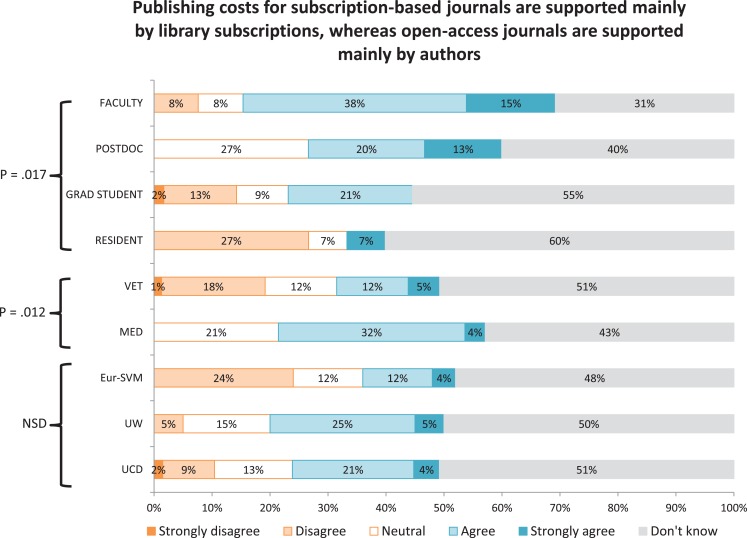
**Differences in levels of agreement based on role, veterinary vs medical audience, and site for survey statement #12**.

A significantly lower proportion of faculty (vs postdocs, graduate students, and residents) indicated that open access was an important factor in deciding where to publish (Figure 2). Higher proportions of faculty and postdocs (vs graduate students and residents) and a lower proportion of respondents from Eur-SVM (compared with other sites) indicated that they expected to pay more to publish in an open-access journal (Figure 3). A higher proportion of respondents from the ASVCP and Eur-SVM (vs UCD and UW) and in a veterinary (vs medical) audience indicated that their article would be cited more frequently if published in an open-access journal (Figure 4). A higher proportion of faculty (compared with other roles) and medical (vs veterinary) respondents agreed with the statement on how publishing costs were supported for subscription-based and open-access journals (Figure 5).

More than half of graduate students (34/57, 60%) and postdocs (9/17, 53%) agreed or strongly agreed that open-access and subscription-based journals upheld similar ethical standards, whereas 52% (11/21) of faculty and 48% (13/27) of residents indicated that they did not know (*P* = 0.0230); several respondents commented that it depended on the journal. More respondents from UCD (42/67, 63%) and Eur-SVM (16/26, 62%) compared with those from UW (8/20, 40%) (*P* = 0.046) agreed or strongly agreed that interested readers would have access to their article regardless of whether it was published in an open-access journal.

**Figure 6:**
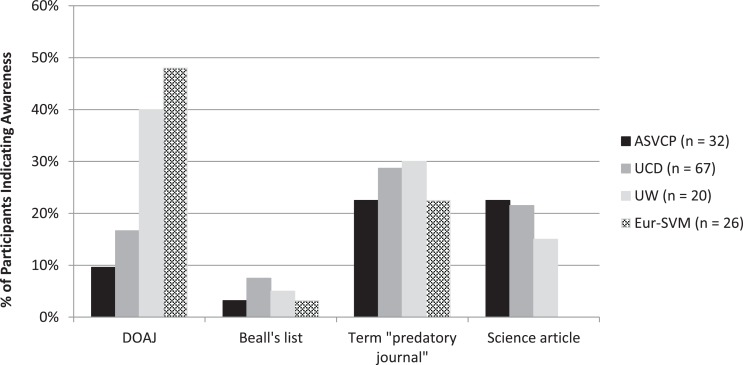
**Percentage of respondents indicating awareness of the Directory of Open Access Journals (DOAJ), Beall’s list, the term “predatory journal”, and the *Science* “sting” article about predatory journals**. Results differed significantly (*P* < 0.03) by site for all questions except awareness of Beall’s list.

Thirty-four of 142 (23.9%) respondents were aware of the DOAJ; 7/143 (4.8%) were aware of Beall’s list, 33/143 (23.0%) were aware of the term “predatory journal”, and 24/142 (16.9%) were aware of the *Science* article about predatory journals. Significant differences in awareness were observed based on site (Figure 6), and a higher proportion of medical (11/28, 39.2%) vs veterinary (20/104, 19.2%) respondents was aware of the term “predatory journal” (*P* = 0.0329). Awareness did not differ significantly based on role.

Of the 145 respondents, 116 (80.0%) defined the term “predatory journal” (Table 2). Respondents were faculty (18, 15.5%), postdocs (16, 13.7%), graduate students (46, 39.6%), residents (22, 18.9%), and other (14, 12.0%). Thirty of 116 (25.8%) respondents had indicated that they were aware of the term “predatory journal.” Responses were summarized and categorized by theme according to criteria for predatory journals; responses containing multiple themes were divided and listed in more than one category for a total of 144 definitions (11). Twenty-two of 144 (15.3%) definitions fit the criteria for predatory journals; of these, 8/22 (36.3%) were by respondents who had indicated awareness of the term “predatory journal.” The majority of definitions (93/144, 64.5%) focused on journal practices considered poor but not predatory: aggressive solicitation of authors with spam email, substandard peer review, and journals whose primary goal is financial gain without regard for scientific quality or ethical standards. Of these definitions, 36/93 (38.7%) were by respondents who had indicated awareness of the term “predatory journal.” Twenty-nine of 144 (20.1%) definitions comprised themes unrelated to predatory journals, including high-quality journals; all these definitions were by respondents who had not previously indicated awareness of the term “predatory journal.”

**Table 2:** **Definitions of “predatory journal” by participants in scientific writing workshops, categorized according to theme**.

Practices considered as predatory^a^	Definitions given by respondents for predatory journals (No.)
*Editor and editorial board lack legitimacy*: unqualified, concocted, appointed without permission or knowledge, exempt from editorial contributions	Hard to pinpoint responsible leaders (1) Editors or website may not even be affiliated to journal (1) Entice big name scientists to lend name (only) to editorial board (1) Essentially the reviewers are fake as well as the editorial board (1)

*Publishing operations lack transparency and legitimacy*: hidden fees, lack of standard policies or practices for digital preservation, indexing, searches	A journal that does not exist, is a scam, is not legit (2) Unscrupulous journals that do not uphold business standards (1) Authors led to submit material, then left paying for it all (1)

*Lack integrity*: journal title does not reflect mission or origin; false claims about impact factor, international standing, indexing; spam requests to unqualified peer reviewers; repeated plagiarism, other ethical breaches	Journals solicit manuscripts under false pretenses (4) Low-quality journal trying to get submissions by marketing techniques (1)

*Other*: republish papers without attribution; operate in a Western country but function mainly as vanity press for scholars in a developing country; minimal or no copyediting or proofing; publishes non-academic papers; hidden contact information	Reprint papers published in other journals, without permission (4) Offer authors incentives; claim to be better than other journals (3) Editorial board has specific agenda to publish articles from certain groups of researchers (1) Hide behind offline-looking operations, hard to pinpoint location (1)

**Poor practices, but not considered predatory**^a^	

Journal excessively broad in scope or combines fields not normally grouped	Journal lacks specific scientific focus (1)

Excessive spam mail to solicit manuscripts or editorial board memberships	Journals that aggressively or indiscriminately solicit authors, e.g., with email or spam (20)

Prominently state promise for unusually rapid peer review and publication	Offers immediate publication of any work (1)

Evidence that the journal does not really conduct a bona fide peer review	Journals that solicit, accept, and publish manuscripts without review, with substandard review or without regard for scientific quality and accuracy (10) Journals that publish poor quality works rejected by respected journals (2)

Publisher appears to focus exclusively on billing and procuring the article processing fee, while not providing services for readers or not making an effort to vet submissions; optional “fast-track” fee-based service that appears to provide assured publication with little or no vetting; entrepreneurial behavior rises to the level of sheer greed, oblivious to business ethics	Journal whose primary goal is to obtain high article fees for financial gain, without regard for scientific or ethical standards (45) Journals that solicit and charge high fees to authors but then do not publish the paper or give it exposure or make it easily available (6) A less-than-qualified, disreputable, low tier, or unknown journal whose primary goal is profits at the expense of scientific or ethical standards (4) Journal’s primary focus is profit through high publication fees (3) Journal publishes manuscripts rejected by others for high fees (1)

**Other themes**

Takes advantage of authors	A journal that preys on or takes advantage of new or inexperienced authors (7) A journal that targets authors with unkept promises of publication or compensation (2) Journals that try to publish studies ± the author’s agreement (1) Journals that do not look at all submitting authors fairly (1) Journals that shut down ideas and results of submitted articles (1)

Takes advantage of other publishers	Journal that makes a profit out of preying on publishers (1) Actively seeks manuscripts to prevent other journals from publishing (1) A “reputable” journal that seeks to discredit another journal (1) A journal that commercially encroaches on existing journals (1) An organization seeking to collect information for less-than-honorable purposes (1)

A high-quality journal	A strict, good quality journal with a high impact factor; first choice for authors (4) Editors ask or invite researchers/authors to write for their journal (3) Journal request papers on new topics or papers that will improve their standing (3) A journal that rejects a large percentage of submissions; difficult to get accepted (2)

^a^Criteria adapted from Jeffrey Beall, 3rd edition, January 1, 2015. Readers are referred to the website for a complete and detailed list of criteria.^1^

## Discussion

Based on our survey results, many or most prospective authors in this cohort, consisting largely of trainees, were unaware of predatory journals or of potential differences among journal models that may be important when selecting a journal for publication. Educational goals that should be included when conducting workshops or mentoring novice authors in scientific writing are to: (1) increase awareness of predatory journals, (2) distinguish among legitimate and illegitimate open-access journals, (3) understand the similarities and differences between open-access and subscription-based journals, (4) learn to evaluate journals and their processes, and (5) select the best journal for the scientific study. Our survey did not explicitly address respondent experience with publishing, an aspect that could be included in future surveys, and responses may have been influenced by lack of publishing experience, especially among Eur-SVM respondents who likely had the least prior experience. We should engage prospective authors in discussions about where to submit their work, but also include experienced faculty and other mentors in these discussions. Sensitivity to and awareness of cultural and geographic considerations for publication are important.

Predatory journals are already numerous and their number is increasing.^1^ Prospective authors should be aware of their existence, but also avail themselves of resources that provide information about these journals. Increased awareness of predatory journals and available resources is needed across countries, institutions, and individual roles; inexperienced authors and those in some geographic regions may be especially vulnerable owing to pressures to have a manuscript accepted, no matter the quality of the journal (8). More than half of the graduate students and postdocs in our survey agreed or strongly agreed that open-access and subscription-based journals upheld similar ethical standards, whereas about half of the faculty and residents indicated that they did not know. A few universities, libraries, and journals provide guidelines for avoiding predatory journals, but until authors recognize the need to proactively seek information, the role of mentors or formal courses and workshops will be important in raising awareness. Naiveté about predatory journals, whose sole goal is profit, was indicated by the high percentage of our respondents who expected that payment of publication charges would not influence the decision to accept a manuscript. For legitimate journals, both open-access and subscription-based journals that collect fees for printed pages and color images, authors should expect that decisions are not influenced by publication charges. Whether or not they were aware of predatory journals, many respondents defined the term as acquisition of fees irrespective of scientific quality or ethical standards, and some respondents asserted that aggressive or indiscriminate email solicitation might indicate that a journal is predatory.

We intentionally limited the survey period to one year after publication of the *Science* article on predatory publishers and journals (which referenced both Beall’s list and the DOAJ), so that the question about awareness of that article remained relevant. Eur-SVM respondents, who had no awareness of the *Science* article, comprised a small and homogeneous group of graduate (Master’s) students who had recently completed their studies in veterinary medicine and had the least experience in publishing compared with other groups; their workshop also was held later than other groups (farthest date from publication of the *Science* article) and they were geographically distant from the U.S., so media exposure to the *Science* article was likely less. For all respondents, few if any identified the criteria defined by Beall, and some completely misunderstood the term “predatory journal.” Even respondents who were aware of the DOAJ had little awareness of Beall’s list, which may have resulted from the ease of finding the DOAJ using the search term *open-access journal*.

Even with increased awareness, educating prospective authors about how to distinguish among legitimate and illegitimate open-access journals is still required. Few respondents across site, role, or field of medicine were aware of Beall’s list, and less than 50% were aware of the DOAJ or the *Science* article. Of course, even these resources do not guarantee identification of journals with legitimate practices; for example, in the published sting operation, journals that accepted the fictitious article included journals listed in the DOAJ and other indexes (9). Thus, although these resources provide useful information and authors ought to be familiar with them, authors should consult with others who have experience publishing in their field and should also critically evaluate articles published in various open-access journals. Additionally, authors can ascertain if the publisher or journal is a member of COPE and if the publisher is recognized as a member of the Open Access Scholarly Publishers Association (OASPA).^4^

The overall goal of publication is to benefit science by making high-quality research accessible to everyone. Thus, both authors and readers desire rigorous peer review to ensure – to the extent possible – scientific quality. A 2014 open-access survey conducted on behalf of Taylor & Francis had little overlap with questions in our survey, but did find that 35% of respondents agreed or strongly agreed that open-access journals were of lower quality than subscription-based journals (12), higher than the 20% of respondents in our survey who disagreed that open-access and subscription-based journals were of similar quality. Peer-review processes can be identified by browsing a journal’s website or guidelines to authors, discerned through direct or indirect experience with a journal, and surmised by critically evaluating the quality of articles published in the journal of interest. Because of the proliferation of predatory and other online journals that lack standards for scientific quality, the peer-review system of subscription-based journals often has more credibility among some authors; this has been disputed, and the sting article in *Science* has been criticized for not including subscription-based journals as a control arm of the study (9). Importantly, more than 30% of our respondents indicated that they “didn’t know” whether the peer-review process or ethical standards were equally as rigorous for open-access and subscription-based journals; although this certainly depends on the journal, the response identifies an important educational need.

Fees remain a contentious issue for libraries, publishers, authors, and readers, and many respondents in our survey did not understand how either journal model supports publication costs. Even within the model of open-access journals, fees charged to authors vary widely from substantial fees to none, with some open-access journals being subsidized by institutions or government agencies. Increasingly, agencies are requiring that funded research be published in open-access journals, making identification of legitimate journals with sound editorial policies even more important. In our survey, expectations for higher author fees when publishing in an open-access journal varied both by role, with faculty and postdocs expecting to pay more, and by site, with more Eur-SVM respondents not expecting to pay more; the latter result was likely attributable to the relative inexperience of the students. Although subscribers understand the fees charged by subscription-based journals, whether for individual subscriptions or those associated with membership in an organization, few are aware of the hefty fees charged to libraries, such as university or institutional libraries, for the same subscription; faculty, staff, and students have free access to those journals without understanding the costs to the university or institution.

Wide access and speedy publication by online open-access journals is considered an advantage, but caution is advised if speed of publication is prioritized over the quality of peer review and editing of the article. Many subscription-based print journals now publish articles online ahead of their appearance in the print journal, and e-publication is considered official. With regard to citation frequency based on type of journal (open-access vs subscription-based), our respondents did not differ in their opinions by role, but those from veterinary medicine had a much higher expectation of more frequent citation of an article published in an open-access journal than did respondents from medicine, and there were major differences of opinion based on site. The Taylor & Francis survey (12) found that 29% of respondents agreed or strongly agreed that their open-access papers would be cited more heavily, as compared with >40% in our survey, where it also varied significantly by discipline (medical vs veterinary) and geographic location. The higher proportion in our survey also could reflect the predominance of graduate students and residents in our study (59% of respondents) compared with the Taylor & Francis survey, in which only 9% of respondents self-identified as PhD, MS, or undergraduate students (12). Nevertheless, data are conflicting on citation advantages. Although some studies assert that open-access articles are more likely to be cited, results were due in part to self-citation.^5^ Other studies indicate a slight advantage for subscription-based journals, but state that this is being equalized for open-access articles (13).

A limitation of our survey was the relatively small sample size; however, nearly 100% of all participants in our workshops and courses responded. In fact, the sample size does not differ much from that of medical disciplines in the Taylor & Francis survey (*n* = 226, 4% of the total), which was large and multidisciplinary, although veterinary medicine was not mentioned (12). Because our survey was used as part of each workshop itself, the face-to-face format was important; furthermore, respondents were unable to search for answers to questions or use online resources, making their responses a true reflection of their current awareness. We acknowledge that the addition of two questions after the ASVCP workshop could have influenced responses to other questions in the survey.

Regardless of the publishing model, authors must learn to evaluate journals based on a wide variety of aspects, including editorial oversight (journal editors and editorial board members), peer-review practices, quality of published articles, access and indexing, metrics and citations, costs, and, importantly, ethical practices. Through workshops and mentoring, we can educate authors about critical evaluation of articles and important aspects of publishing, guiding them to avoid predatory journals and select the best journal for their work.

## Conflict of Interest Statement

The survey and course materials in this study were developed without financial support. The authors were reimbursed for some travel expenses for two of the workshops. The workshop at UC Davis School of Veterinary Medicine was supported by a grant from the Virginia Perry Wilson Endowment. The workshop at UC Davis School of Medicine was supported by the National Center for Advancing Translational Sciences, National Institutes of Health, through grant number UL1 TR000002; the content is solely the responsibility of the authors and does not necessarily represent the official views of the NIH. Mary Christopher coordinates the International Association of Veterinary Editors, which receives sponsorship from Wiley and Elsevier. Mary Christopher is the Field Chief Editor of *Frontiers in Veterinary Science*; she was not involved in the peer review process or the decision to publish this manuscript.

## Footnotes

^1^Scholarly Open Access, http://scholarlyoa.com

^2^Directory of Open Access Journals, http://doaj.org

^3^Committee on Publication Ethics, http://publicationethics.org

^4^Open Access Scholarly Publishers Association, http://oaspa.org/membership/members/

^5^OpCit Project, http://opcit.eprints.org/oacitation-biblio.html
